# Personality traits and high cigarette dependence among university students: Insights from Lebanon

**DOI:** 10.1371/journal.pone.0298193

**Published:** 2024-02-15

**Authors:** Imad Bou-Hamad, Jaafar Hoteit, Nadine Yehya, Lilian Ghandour

**Affiliations:** 1 Department of Business Information and Decision Systems, Suliman S. Olayan School of Business, American University of Beirut, Beirut, Lebanon; 2 Department of Public Affairs and Marketing, University of California, Davis, California, United States of America; 3 Department of Epidemiology and Population Health, Faculty of Health Sciences, American University of Beirut, Beirut, Lebanon; Karnali Academy of Health Sciences, NEPAL

## Abstract

**Purpose:**

The use of tobacco and cigarette products remains widespread globally, with varying patterns across countries. Understanding the factors influencing cigarette dependence among young adults is crucial for effective smoking prevention and control programs. Personality traits are one of the factors that influence smoking behaviour, yet the evidence on their role in high cigarette dependence among young adults remains inconclusive. This study aims to provide insights and initial evidence on the potential association between personality dimensions, sociodemographic factors, lifestyle habits, and high cigarette dependence among Lebanese university students.

**Methods:**

A convenient sample of 212 student smokers from one private and one public university in Lebanon participated in an online survey. The survey included measures of personality traits using the Big-Five framework, sociodemographic factors, lifestyle habits, and the Fagerström Test for Cigarette Dependence (FTCD). Logistic regression models and mediation analysis were used to analyze the data.

**Results:**

The results revealed significant associations between personality dimensions and high cigarette dependence among Lebanese university students. Smokers with higher levels of Openness to Experience were more likely to have high cigarette dependence (β = 0.408, p < = 0.015). Conversely, smokers with higher levels of Conscientiousness (β = -0.500, p < 0.001) and Agreeableness (β = -0.491, p < 0.01) were less likely to have high cigarette dependence. Additionally, attending a public university (β = 1.198, p = 0.018), having more close friends who smoke (β = 0.525, p < 0.01), and switching to a cheaper cigarette brand (β = 0.928, p < 0.05) were associated with a higher cigarette dependence.

**Conclusion:**

These findings highlight the importance of considering personality dimensions, sociodemographic factors, and lifestyle habits in understanding high cigarette dependence among Lebanese university students. The results can inform the development of targeted interventions to address high cigarette dependence in this population.

## Introduction

Globally, the World Health Organization (WHO) estimates that tobacco use results in more than 8 million deaths annually. More than 7 million of those deaths are the result of direct tobacco use, and around 1.2 million from non-smokers being exposed to secondhand smoke. While tobacco and cigarette product usage persists worldwide, its prevalence varies based on geographic and demographic factors. While tobacco use is decreasing in some developed countries, it is increasing in developing countries [1]. Also, young adults, disadvantaged individuals, and women show less declines in smoking behavior [1]

Providing a global, regional, and local perspective on cigarette use and dependence is crucial for a nuanced understanding of this complex issue. Recent studies have shown that nicotine alone cannot account for cigarette or smoking addiction. Other factors, such as the behavior of smoking itself, also contribute to the development of cigarette dependence [2]. Smokers with a high susceptibility to cigarette dependence may have a harder time quitting smoking. Nicotine withdrawal symptoms, such as anxiety, depressive-like behavior, and difficulty concentrating, can be severe and make quitting smoking challenging [3].

Distinguishing cigarette dependence as a distinct concept from other metrics of tobacco consumption, such as smoking frequency or quantity, provides a better insight into tobacco use behavior. This differentiation enhances the evaluation of the efficacy of smoking prevention and control programs [4]. Studies have indicated that individuals with high dependence are more likely to use other substances, including alcohol, cannabis, and cocaine, potentially indicating an underlying tendency towards psychopathology [5]. Therefore, it is crucial to study high cigarette dependence to identify an individual’s susceptibility to developing other substance use disorders or psychiatric morbidity. This knowledge can help in designing targeted interventions to address this issue effectively.

While previous studies have investigated factors influencing smoking status, smoking progression, and cigarette dependence among adults and the general population, much less is known about the influence of personality dimensions on high cigarette dependence among young adults, particularly during times of economic and social distress. Despite the growing research interest in understanding the personality influences on smoking behavior [6], the published evidence remains inconclusive. The Big-Five framework, also known as the Five-Factor Model (FFM) is recognized for its widespread acceptance, serves as a robust foundation for the assessment of personality traits. It is a cross-culturally validated model that encapsulates a multifaceted view of human personality, encompassing five distinctive dimensions.

Firstly, Extraversion probes into attributes associated with sociability, the nurturing of positive emotions, and a proclivity for the pursuit of excitement in one’s experiences. Secondly, Openness to Experience provides insight into qualities such as curiosity, imagination, and a profound inclination to embrace novel and unconventional encounters. Thirdly, Agreeableness engulfs traits characterized by compassion, empathy, modesty, and a thoughtful disposition toward others. Fourthly, Conscientiousness delves into qualities like ambition, dutifulness, reliability, and an innate capacity for self-discipline. Finally, Neuroticism scrutinizes emotional sensitivity, stability, and a predisposition toward grappling with anxiety and depressive tendencies, making it a pivotal aspect of personality assessment [7–9].

The exploration of the interplay between personality and smoking is not novel. This area of investigation has a longstanding history, dating back to Eysenck’s work in 1980. Studies have suggested that individuals with high Extraversion may turn to smoking to seek stimulation, while those with high Neuroticism may resort to smoking as a means of tension reduction [10, 11]. Previous research has also unveiled diverse relationships between these dimensions and different facets of smoking behavior. Specifically, individuals scoring higher on Extraversion, Neuroticism, and Openness to Experience have exhibited a heightened propensity toward smoking, while those characterized by elevated levels of Agreeableness and Conscientiousness are less inclined to engage in smoking behavior [7].

Despite the valuable insights from previous research, there is a significant gap in understanding the influence of personality dimensions on high cigarette dependence among young adults, especially within the context of economic and social distress.

Lack of affordability is a factor which could impact individuals’ smoking status and progression. It can deter smokers from cigarette dependence as it incentivizes the behavior of quitting. It may also impact smokers’ cigarette brand loyalty as price changes do not necessarily influence individuals with better purchasing power to quit cigarette use [12].

As individual factors, such as gender, age, education, and various sociodemographic and lifestyle variables, can serve as moderating elements in the relationship between personality and smoking behavior, our study offers a comprehensive perspective on the intricate interplay between personality dimensions and the phenomenon of high cigarette dependence.

it investigates how the personality traits, lifestyle habits, and sociodemographic factors, and cigarette brand switching behavior associate with high cigarette dependence among Lebanese young adults in a context of ongoing economic and social distress. Lebanon is a developing country that still has one of the highest cigarette smoking prevalence estimates in the Arab region [13].The country has been dealing with a severe economic recession, poverty, and a rapid currency devaluation crisis since 2019.

## Methodology

### Study design

This study received ethical approval from the Institutional Review Board (IRB) at the American University of Beirut (AUB), underscoring the adherence to established research ethics. We conducted a rigorous cross-sectional study with the primary objective of investigating the multifaceted factors contributing to high cigarette dependence among university students in Lebanon. To ensure a comprehensive representation of the student population, our study targeted both undergraduate and graduate students from two distinct universities in Lebanon. These institutions, namely the American University of Beirut (AUB), a prestigious private university, and the Lebanese University (LU), the sole public university in Lebanon, were strategically selected to encompass a wide socio-economic spectrum. LU caters to a diverse student body, inclusive of individuals from low-income backgrounds and rural regions, while AUB predominantly serves a more affluent student demographic. This deliberate selection of universities enhances the study’s ability to capture a broad range of perspectives and socio-economic factors that may influence cigarette dependence among university students in Lebanon. The sampling method used in this study was convenience sampling. While this method does not allow drawing general conclusions, it can still be valuable in indicating potential relationships between variables [14].

### Participants and data collection

To determine our inclusion criteria, we ensured that participants who hadn’t smoked in the past 30 days were excluded from the survey. Our study focused on graduate and undergraduate students from AUB or LU between the ages of 18 and 30 who had smoked at least one cigarette in the past month.

The survey was distributed by e-mail to undergraduates and graduate students registered at AUB and LU. Data was collected from February 25 to March 31, 2022. The participants were provided with the objectives, details, and written informed consent form of this study through an e-mail recruitment text and on the first page of the survey. They were asked to sign the online consent form before starting the survey. Moreover, participants did not receive any financial incentives, and all data collected was anonymized to safeguard confidentiality and ensure data reliability. To avoid double sampling, we employed specific strategies for AUB and LU students. AUB students received survey invitations via email, and once they completed the survey through the provided link, access was restricted to prevent multiple submissions. For LU students, we collaborated with department heads to distribute survey links, and the same access restriction measures were applied. To ensure the robustness of our study, we initiated a comprehensive sample size of 1,200 individuals to whom the survey was distributed. Of this pool, 622 respondents participated, resulting in an initial response rate of 51.8%. To uphold data integrity, stringent screening measures were applied, including the exclusion of records with missing data fields. Following this meticulous process and confirming adherence to predefined inclusion criteria, a final cohort of 212 individuals emerged, representing an adjusted response rate of 17.6%. This cohort comprised 126 males and 86 females, with an overall mean age of 22.54 years. Within this gender-stratified breakdown, 79 males originated from AUB, with a mean age of 22.30, while 47 males hailed from LU, with a mean age of 23.55. Among females, 66 were affiliated with AUB, with a mean age of 21.90, and 20 were associated with LU, exhibiting a mean age of 23.25.

It is crucial to emphasize that our study was conducted with unwavering adherence to the ethical guidelines governing human subjects’ research, as stipulated in the Declaration of Helsinki.

### Measures

The study’s variables fall into three distinct clusters: personality traits based on the Big Five model, sociodemographic and lifestyle factors, and the variable measuring smoking dependence.

#### Personality traits

*Openness to experience.* People who score high on this personality trait are creative, curious, and open-minded, while those who score low are more focused on routine and tradition [15]. A study by Zvolensky et al. (2015) revealed that elevated levels of openness to experience were significantly linked to increased risk of any lifetime cigarette use [16]. Furthermore, based on a 2013 study, Openness to Experience was found to play a key role in long-term quitting behaviors among Chinese smokers following treatment [17]. Consequently, we tested the following hypothesis:

**H1:** Young adults with elevated levels of Openness to Experience are more likely to have high cigarette dependence.

*Conscientiousness.* People with high Conscientiousness levels are often diligent, responsible, and reliable [15].There is no universal agreement on the direction of Conscientiousness’s influence on smoking behavior. While some studies found a positive association between high levels of Conscientiousness [18, 19] others found a negative association [16–20].

Although the literature has produced some inconsistent findings on the relationship between Conscientiousness and smoking behavior, the existing evidence implies that individuals with higher levels of Conscientiousness tend to avoid the leading behavioral contributors to mortality, such as smoking [21], and thus we tested the following hypothesis:

**H2:** Young adults with elevated levels of Conscientiousness are less likely have high cigarette dependence.

*Extraversion.* Those who score high on this trait tend to be talkative, confident, and energetic [15]. A 2007 study revealed that smokers had higher levels of Extraversion [6]. Moreover, a more recent meta-analysis of nine cohort studies concluded that current smoking and smoking initiation among adults were associated with higher Extraversion [20]. It concluded that adult smokers tend to have elevated levels of Extraversion than non-smokers. Therefore, we examined the following hypothesis:

**H3:** Young adults with elevated levels of Extraversion are more likely to have high cigarette dependence.

*Agreeableness*. Individuals who score high on Agreeableness tend to be trusting, friendly, and have a desire to help others. In a study conducted by Choi et al. (2017), it was found that European American smokers exhibited higher levels of Agreeableness compared to non-smokers. The study also found that among African Americans, higher levels of Agreeableness were associated with an increased likelihood of being a current smoker [19]. Therefore, we tested the following hypothesis:

**H4:** Young adults with higher levels of Agreeableness are more likely to have high cigarette dependence.

*Neuroticism (emotional stability)*. Individuals high in Neuroticism tend to experience less Emotional Stability and are more likely to perceive situations as stressful. The Ten Item Personality Inventory (TIPI), which is a set of questions used to measure the personality dimensions of participants, refers to Neuroticism as Emotional Stability.

Studies have shown that Neuroticism positively predicts smoking relapse, is negatively linked to smoking cessation and increased risk of lifetime cigarette use and is a significant predictor of current smoking status [6, 16, 20]. Hence, we evaluated this hypothesis:

**H5:** Young adults with low levels of Emotional Stability are more likely to have high cigarette dependence.

The TIPI scale was used to assess the personality dimensions of the participants. It is a brief 10-item measure of the Big-Five framework (Five-Factor Model). Each personality dimension (Extraversion, Agreeableness, Conscientiousness, Emotional Stability, and Openness to Experience) was assessed using two items, on a 7-point Likert scale. To calculate the score for each dimension, the average of the two items were calculated, resulting in a score between 1 and 7.

The TIPI has reached adequate levels of convergence with the widely used Big-Five framework measures in test-retest reliability and patterns of predicted external correlates [15]. The internal consistency of the personality dimensions is as follows: Extraversion (α = 0.58), Agreeableness (α = 0.35), Conscientiousness (α = 0.5), Emotional Stability (α = 0.48), and Openness to Experience (α = 0.4). When considering the TIPI scale as a comprehensive tool that integrates these dimensions into a unified assessment, the overall internal consistency is computed at a respectable α = 0.56.

It should be noted that TIPI was designed to measure very broad domains with only two items per personality dimension at both the positive and negative poles [15].

#### Sociodemographic factors and lifestyle habits

At the end of the survey, participants were asked about their lifestyle habits and sociodemographic characteristics. This included their age, gender, education level, income sufficiency, employment status, marital status, smoking behavior, and more. It’s worth noting that respondents were also prompted to categorize their income into five distinct levels: ’Very low income,’ indicating an insufficiency to cover basic needs for a month; ’Low income,’ denoting income that barely covers monthly needs; ’Medium,’ reflecting income sufficient for all basic needs; ’High,’ signifying income that not only covers necessities but allows for a few luxury items; and ’Extremely high,’ applicable to income levels that cover a wide range of luxury items. This detailed classification aims to capture participants’ perceptions regarding the sufficiency of their income, providing valuable insights into the socioeconomic context of our study.

#### Fagerstrom Test for Cigarette Dependence (FTCD)

The Fagerstrom Test for Cigarette Dependence (FTCD), originally named Fagerstrom Test for Nicotine Dependence (FTND), was used to assess participants’ cigarette dependence. Two items on the FTCD were on a 4-point scale, and four items were on a 2-point scale. To calculate the cigarette dependence for each participant, the six items in FTCD were added up to provide a score between 0 and 10 [22].Smokers who scored below 6 were considered to have low to moderate cigarette dependence, and those who scored 6 and above were to have high cigarette dependence. The cut-off point of 6 in the FTND/FTCD for classifying individuals as those with and without high nicotine dependence is considered as the ‘‘gold standard” [23]. It should be noted that smokers who receive a score of 0 on the FTCD are either not dependent on nicotine or have a very low-level dependence [22].

The FTCD was validated on the Lebanese population [24–26]. In this study, the FTCD achieved an internal consistency of (α = 0.8).

### Data analysis

To rigorously analyze our dataset and derive meaningful insights, we employed a diverse array of statistical tests and techniques using the R programming language.

We employed descriptive statistics using the R package **Tidyverse**. This facilitated the concise summarization of sociodemographic characteristics and lifestyle habits, painting a clear picture of the participants’ diverse profile.

For hypothesis testing, particularly in the investigation of the five hypotheses pertaining to the correlation between personality traits and high cigarette dependence, we leveraged logistic regression models. The standard, built-in **stats** package in R served as the foundation for executing these models. Logistic regression, a powerful tool, allowed us to scrutinize the connections between individual personality traits and the probability of high cigarette dependence, delivering valuable insights into the potential impact of personality on smoking behavior.

To gauge the internal consistency of the personality dimensions gauged by the Ten Item Personality Inventory (TIPI), we turned to the R package **psych**. This pivotal tool facilitated the calculation of Cronbach alpha scores, providing a reliability metric for the assessed personality dimensions.

Moreover, mediation analysis was performed to investigate potential indirect pathways through which these personality traits influence cigarette dependence, mediated by sociodemographic or lifestyle variables. For this purpose, we employed **Bayes Process** (Model 4). While not a standalone package, Process for R is a user-defined function, and we obtained the necessary R file from the following website (https://www.processmacro.org/download.html) for the mediation analysis. This approach allowed us to investigate potential pathways through which personality traits might exert influence on cigarette dependence.

## Results

### Descriptive statistics

The sample consists of 212 smokers, with 59.43% of them being males (According to Table 1). Regarding employment status, a significant portion, 52.36% of respondents, report being unemployed, while the remaining 47.64% are employed. In terms of marital status, the majority are singles (81.13%), with smaller proportions being engaged (6.13%), married (4.72%), divorced or separated (1.89%), or in a relationship (6.13%). When it comes to perceptions of income sufficiency, 40.09% consider their income as "medium," 33.49% perceive it as "high," and 19.34% categorize it as "low."

**Table 1:** Demographic and lifestyle characteristics of study participants.

Variable	Mean	SD	Percent. (%)
**Gender**	-	-	F (40.57)
			M (59.43)
**Age**	22.55	2.87	-
**Employment**	-	-	Employed (47.64)
			Unemployed (52.36)
**Marital Status**	-	-	Single (81.13)
			In a relationship (6.13)
			Engaged (6.13)
			Married (4.72)
			Divorced (1.89)
**Income Sufficiency**	-	-	Extremely High (2.36)
			High (33.49)
			Medium (40.09)
			Low (19.34)
			Very low (4.72)
**Household Members**	4.10	1.67	-
**Social Media Use**	-	-	1–2 Hours (25.47)
			2–3 Hours (39.15)
			3–4 Hours (15.09)
			Less than 1 Hour (3.77)
			More than 4 Hours (15.09)
			Doesn’t use (1.42)
**Exercise Frequency**	-	-	Never (29.25)
			Sometimes (37.26)
			Often (25.47)
			Every day (8.02)
**Cigarette Brand Switching (due to crisis)**	-	-	Did not switch brand (63.21)
			Switch to cheaper brand (34.91)
			Switch to more expensive brand (1.89)
**Close friends who smoke**	3.15	1.47	-
**Current Smoking Behavior**	-	-	Decreased smoking (21.33)
			Increase smoking (36.32)
			Same amount (42.45)

In terms of social media usage, the majority (39.15%) spend between 2 to 3 hours daily on social media, while 25.47% allocate 1 to 2 hours, and 30.18% dedicate over 3 hours. As for exercise patterns, 37.26% report sometimes exercising, 29.25% engage in no exercise, 25.47% exercise often, and 8.02% exercise daily or at least five times a week.

Furthermore, during Lebanon’s economic crisis and revolution in 2019, 63.21% of respondents retained their preferred cigarette brand, while 34.91% switched to a more affordable alternative, and 1.89% opted for a more expensive one. Concerning changes in smoking behavior during this period, 42.45% reported no change, 36.32% increased their daily cigarette consumption, and 21.23% reduced it. Additional insights include the average number of close friends who smoke (mean: 3.15) and household members (mean: 4.10), as well as the mean age of respondents (22.55) with a standard deviation of 2.87.

### Logistic regression analysis

Two logistic regression models were run with cigarette dependence (FTCD) as a binary outcome reflecting high dependence. The first model included only the five personality dimensions as the independent variables; the second model further controlled for sociodemographic and lifestyle variables as independent variables. The unadjusted results are presented in **Table 2**. It is crucial to emphasize that, for the sake of interpretability, odds ratios below 1 discussed in the following sections are presented in inverted form. This practice is standard in logistic regression.

**Table 2:** Logistic regression output for exploring impact of personality dimensions on cigarette dependence.

	Beta	SE	Z Value	Pr(>|z|)	Odds Ratio	CI
Intercept	1.492	1.283	1.163	0.244	4.448	[0.360; 54.964]
Extraversion	0.156	0.114	1.365	0.172	1.169	[0.935; 1.461]
Agreeableness	-0.491	0.156	-3.142	0.001 **	0.611	[0.451; 0.831]
Conscientiousness	-0.500	0.138	-3.619	0.0002 ***	0.605	[0.463; 0.795]
Emotional Stability	-0.113	0.121	-0.932	0.351	0.892	[0.705; 1.132]
Openness to Experience	0.408	0.168	2.427	0.015 *	1.505	[1.082; 2.090]

Beta(s) are regression coefficients, SE(s) are standard errors

* p-value < 0.05

** p-value < 0.01

*** p-value< 0.001, CI(s) denote the 95% confidence intervals for the odds ratios.

As can be seen, levels of Openness to Experience, Conscientiousness, and Agreeableness are significant predictors of high cigarette dependence.

Young adults with higher levels of Openness to Experience are more likely to have high cigarette dependence (beta = 0.4088, p < 0.05), which validates hypothesis **H1**. The odds of having high cigarette dependence are 1.5 times higher for every 1 unit increase in Openness to Experience.

In addition, more Conscientious smokers are less likely to have high cigarette dependence (beta = -0.5009, p < 0.001), which supports hypothesis **H2**. The odds of having high cigarette dependence are 1.65 times lower for every 1 unit increase in Conscientiousness.

Moreover, more agreeable smokers are less likely to have high cigarette dependence (beta = -0.4916, p < 0.01), which rejects hypothesis **H4**. The odds of having high cigarette dependence are 1.63 times lower for every 1 unit increase in Agreeableness.

Hypotheses **H3** and **H5** were not supported because Extraversion and Emotional Stability were not significant predictors (p > 0.05) of high cigarette dependence in this study.

The adjusted results controlling for sociodemographic and lifestyle factors are presented in **Table 3**.

**Table 3:** Logistic regression model for exploring impact of personality dimensions on cigarette dependence after adjusting for sociodemographic and lifestyle variables.

	Beta	SE	ZValue	Pr(>|z|)	Odds Ratio	CI
Intercept	-2.729	1.973	-1.383	0.166	0.065	[0.001; 3.121]
Extraversion	0.158	0.145	1.087	0.276	1.171	[0.881; 1.556]
Agreeableness	-0.575	0.195	-2.942	0.003 **	0.562	[0.384; 0.825]
Conscientiousness	-0.613	0.192	-3.182	0.001 **	0.541	[0.372; 0.789]
Emotional Stability	0.017	0.167	0.105	0.916	1.017	[0.733; 1.411]
Openness to Experience	0.223	0.205	1.089	0.276	1.250	[0.836; 1.868]
Education Sector (Public)	1.198	0.509	2.353	0.018 *	3.313	[1.222; 8.986]
Gender (Female)	0.543	0.460	1.181	0.237	1.722	[0.699; 4.240]
Employment Status (Employed)	-0.239	0.423	-0.565	0.572	0.787	[0.344; 1.804]
Marital Status (Engaged)	0.538	0.835	0.645	0.518	1.713	[0.333; 8.799]
Marital Status (Married)	-0.831	1.120	-0.743	0.457	0.435	[0.048; 3.913]
Marital Status (Divorced/Separated)	3.501	1.788	1.957	0.050	33.148	[0.997; 1102.659]
Marital Status (In Relationship)	-0.359	0.851	-0.422	0.673	0.698	[0.132; 3.702]
Smoking Behavior (Increased)	0.377	0.455	0.829	0.406	1.458	[0.598; 3.557]
Smoking Behavior (Decreased)	-1.320	0.680	-1.942	0.052	0.2661	[0.070; 1.013]
Close Friends Who Smoke	0.525	0.159	3.289	0.001 **	1.691	[1.238; 2.309]
Income Sufficiency	0.724	0.274	2.641	0.008 **	2.062	[1.206; 3.529]
Household Members count	0.330	0.136	2.419	0.015 *	1.391	[1.065; 1.816]
Social Media Use (Moderate)	0.054	0.522	0.104	0.917	1.055	[0.379; 2.936]
Social Media Use (Excessive)	-0.164	0.673	-0.245	0.806	0.848	[0.227; 3.174]
Exercise (Sometimes or few days per month)	-1.063	0.500	-2.124	0.033 *	0.345	[0.130; 0.920]
Exercise (Often or at least 3 days a week)	-0.282	0.609	-0.463	0.643	0.754	[0.229; 2.488]
Exercise (Every day or at least 5 days a week)	-0.585	0.798	-0.733	0.463	0.556	[0.117; 2.662]
Switching Behavior: Switching to a cheaper brand alternative	0.928	0.462	2.005	0.044 *	2.530	[1.023; 6.256]

Beta(s) are regression coefficients, SE(s) are standard errors

* p-value < 0.05

** p-value < 0.01

*** p-value< 0.001, CI(s) denote the 95% confidence intervals for the odds ratios.

More Conscientious smokers are less likely to have high cigarette dependence (beta = -0.613, p < 0.001). The odds of having high cigarette dependence are 1.84 times lower for every 1 unit increase in Conscientiousness.

More agreeable smokers are less likely to have high cigarette dependence (beta = -0.575, p < 0.01). The odds of having high cigarette dependence are 1.77 times lower for every 1 unit increase in Agreeableness.

Furthermore, the study showed that those who switch to a cheaper cigarette brand are more likely to have high cigarette dependence (beta = 0.928, p < 0.05). The odds of having high cigarette dependence are 2.53 times higher for those who switch to a cheaper cigarette brand.

Public university smokers are more likely than private university smokers to have high cigarette dependence (beta = 1.198, p < 0.05). The odds of having high cigarette dependence are 3.31 times higher for public university smokers.

Smokers with more close friends who smoke are more likely to have high cigarette dependence (beta = 0.525, p < 0.001). The odds of having high cigarette dependence are 1.69 times higher for every, one additional close friend who smokes.

Smokers with better income sufficiency are more likely to have high cigarette dependence (beta = 0.724, p < 0.01). The odds of having high cigarette dependence are 2.06 times higher for every 1-unit improvement in income sufficiency.

Furthermore, smokers with more household members are more likely to have high cigarette dependence (beta = 0.330, p < 0.05). The odds of having high cigarette dependence are 1.39 times higher for every additional household member.

Finally, smokers who exercise a few days in a month (compared to never) are less likely to have high cigarette dependence (beta = -1.063, p < 0.05). The odds of having high cigarette dependence are 2.89 times lower for smokers who exercise a few days a month (compared to never).

### Mediation analysis of indirect effect on high cigarette dependence

It should be noted that once sociodemographic and lifestyle variables were adjusted for, Openness to Experience was no longer a statistically significant predictor of high cigarette dependence. This is because the effect of Openness to Experience on high cigarette dependence is mediated by the number of close friends who smoke cigarettes. Mediation analysis was implemented using Bayes Process (Model 4 in R Studio) to test the indirect effect of Openness to Experience on high cigarette dependence. **Table 4** provides the results of the indirect effect test from the mediation analysis. Since the indirect effect is bootstrapped, it does not show p-value. Instead, we look at the bootstrap confidence interval. The lower limit of the confidence interval is 0.014, and the upper limit of the confidence interval is 0.289. The confidence interval does not include 0 since both side of the interval are positive, and thus we can conclude that the indirect effect is significant, and mediation exists.

**Table 4:** Indirect effect(s) of openness to experience on cigarette dependence.

	Effect	Boot SE	Boot LLCI	Boot ULCI
Number of Close Friends Who Smoke Cigarettes	0.119	0.071	0.014	0.289

Effect is the estimated indirect effect, Boot SE is the Bootstrap Standard Error, Boot LLCI and Boot ULCI are the Bootstrap Lower and Upper-Level Confidence Intervals, respectively.

## Discussion

Our study revealed that lower levels of Conscientiousness were linked to high cigarette dependence. In other words, smokers who are more careless and disorganized, and less dependable and self-disciplined, are more likely to have high cigarette dependence. Furthermore, our study found that higher levels of Openness to Experience and lower levels of Agreeableness were linked to high cigarette dependence.

This finding aligns with previous research suggesting that smokers tend to exhibit lower levels of Conscientiousness compared to non-smokers [20]. Moreover, previous studies have demonstrated a higher risk of lifetime cigarette use associated with lower Conscientiousness [16]. Additionally, our study found that higher levels of Openness to Experience and lower levels of Agreeableness were linked to high cigarette dependence. While there is limited prior research specifically examining personality dimensions within the Big-Five framework and their relationship with high cigarette dependence, existing evidence indicates that individuals with higher levels of Openness to Experience are more likely to smoke [6, 16, 17, 27]. Furthermore, research has shown that individuals with higher levels of Agreeableness are more likely to be current smokers [19]. Future research could further explore the pathways through which different levels of Conscientiousness, Openness to Experience, and Agreeableness may be associated with cigarette dependence.

It is worth noting the sociodemographic predictors and lifestyle factors associated with high cigarette dependence. The results revealed that smokers from the public university are more likely to have high cigarette dependence than smokers from the private university [28]. The data collected for this study was during a period in which the learning environment was radically changed due to the pandemic and prospect of future jobs reduced due to the economic crisis. It is known that stress and smoking share a cyclic relationship. Stress induces cigarette craving for smokers, which produces the illusion that smoking in turn relieves stress [29]. Thus, future studies should investigate whether stress, anxiety, and the lack of readiness for online learning in Lebanon’s universities contribute to high cigarette dependence among smokers [30].

Additionally, sociodemographic variables which include having more household members and more close friends who smoke are associated with high cigarette dependence. These findings are consistent with existing literature, which suggests that individuals are more likely to be smokers and less likely to quit smoking when they have friends or family members who smoke [30–32]. Moreover, evidence indicates that smokers with more friends who smoke are more likely to have high cigarette dependence [33, 34].

Interestingly, our study deviates from the literature in one aspect: we found that higher income sufficiency is associated with high cigarette dependence, contrary to the conventional wisdom that lower income is linked to higher cigarette consumption and dependence [29, 35, 36]. This unexpected result may be attributed to the significantly reduced purchasing power of lower income households, which could incentivize individuals to reduce their cigarette consumption or quit smoking altogether, thus avoiding the development of cigarette dependence. It is essential to conduct further investigations to gain deeper insights into this intriguing finding [37, 38].

Additionally, the study found that cigarette smokers who exercise sometimes or a few days per month as compared to never exercising are less likely to have high cigarette dependence. This could be linked to how exercise manages cigarette cravings, withdrawal symptoms, smoking behavior, and cessation [39–41].

An intriguing result from our study indicates that smokers who switch to a cheaper cigarette brand are more likely to have high cigarette dependence. Although the literature does not directly establish a connection between switching to a cheaper brand and high cigarette dependence, it does discuss various pathways smokers follow when faced with affordability or price-related challenges, including reducing cigarette consumption, switching to a cheaper brand, or employing a combination of these strategies [12, 42–44].

### Strength and limitations

Despite the strengths of the current study, there are some limitations. First, the convenience sampling method utilized does not guarantee that the results can be generalized to a larger population. However, it can be a valid way to identify possible relationships between variables. To increase the reliability and accuracy of the findings, it is recommended that future studies use probability-sampling methods [14]. Second, participants of this study were limited to students from two universities: AUB and LU. Having participants from other universities may have expanded the scope of this study and provided other conclusions. Still, these two universities are renowned for their student heterogeneity. Third, since our data collection was through an online survey, there is a selection bias which may have impacted our findings. Careless responding and attrition in online surveys introduce measurement error and can lead to several psychometric issues [45]. As a result, the insignificant impact of Extraversion on high cigarette dependence in this study may have been due to this sampling bias.

Furthermore, our study did not include e-cigarette smokers. Future research can test our findings on participants who use e-cigarettes, as e-cigarette use is increasingly becoming popular among young adults.

Lastly, it is essential to emphasize that our study should be viewed as exploratory, and its results are suggestive rather than conclusive. The utilization of convenience sampling in this study can be considered a preliminary step, laying the groundwork for more scientifically rigorous research methodologies. Our study serves as a pilot investigation that can guide and inform future research endeavors.

## Conclusion

The aim of this study is to uncover the personality traits that are implicated in vulnerability to high cigarette dependence. There are substantial studies that have determined that school-based alcohol prevention programs targeting youth with personality risk factors for addiction and mental health problems have been found to prevent tobacco use, reduce substance use and misuse, and prevent onset of alcohol misuse and dependence in those with elevated personality profiles [46–48].While our findings do not provide evidence of successful personality-targeted interventions in preventing high cigarette dependence, our study does uncover some of the vagueness behind the personality dimensions of the Big-Five framework which are most vulnerable to high cigarette dependence. As a result, this study can provide a benchmark on a research level for future studies that aim to further understand the relationship between personality dimensions and high cigarette dependence in the Middle East and on a larger scale.
